# Evaluation of a digital health decision intervention to support management decision-making for adults with hearing loss: protocol for the HearChoice randomised controlled trial

**DOI:** 10.1136/bmjopen-2025-106751

**Published:** 2025-10-23

**Authors:** Melanie A Ferguson, Kerry A Sherman, Ellen Bothe, Barbra HB Timmer, Piers Dawes, Bronwyn Myers, Richard Norman, Jorge Mejia, Rebecca J Bennett, Abigail L Mottershaw, Elena Meyer zu Brickwedde, Elizabeth Convery, Alex Gyani

**Affiliations:** 1 Curtin School of Allied Health, Curtin University, Perth, Western Australia, Australia; 2 Curtin enAble Institute, Curtin University, Perth, Western Australia, Australia; 3 Lifespan Health and Wellbeing Research Centre, Macquarie University, Sydney, New South Wales, Australia; 4 School of Psychological Sciences, Macquarie University, Sydney, New South Wales, Australia; 5 School of Health and Rehabilitation Sciences, University of Queensland, Brisbane, Queensland, Australia; 6 Sonova Holding AG, Stäfa, Switzerland; 7 Alcohol, Tobacco and Other Drug Research Institute, South African Medical Research Council, Tygerberg, South Africa; 8 School of Population Health, Curtin University, Perth, Western Australia, Australia; 9 National Acoustic Laboratories, Sydney, New South Wales, Australia; 10 Behavioural Insights Team, London, UK; 11 Behavioural Insights Team, Sydney, New South Wales, Australia

**Keywords:** Audiology, Decision Making, Digital Technology, eHealth, Health Education, Hearing

## Abstract

**Introduction:**

Hearing loss is highly prevalent and impacts many aspects of a person’s life, including communication, social engagement, employment, general health and well-being. Yet, many people do not access hearing healthcare and are unaware of the range of hearing healthcare options available. Barriers to hearing healthcare include poor understanding of hearing loss and its impact; poor knowledge of help-seeking for hearing healthcare options; minimal support to help decide which option is best; and stigma related to hearing loss. These barriers lead to many people not receiving the hearing healthcare they need. Guided by theories of behaviour change and implementation science, *HearChoice*, an online tailored decision support intervention, has been co-developed to empower adults with hearing difficulties by offering them choice and control over their own hearing healthcare. *HearChoice* aims to facilitate informed decisions, accessibility and uptake of hearing healthcare, including a wide range of interventions, for adults with hearing difficulties. The objectives of the trial are to evaluate the effectiveness, health economics and feasibility of *HearChoice*.

**Methods and analysis:**

This online randomised controlled trial will recruit participants with hearing difficulties across Australia, with an anticipated sample size of 640. Participants will be randomised to either *HearChoice* (treatment) or an Australia-specific Hearing Option Grid (active control), both delivered online. Outcomes will be assessed at baseline when the interventions will be offered, at 7 days post-intervention (primary endpoint) and at 3 months post-intervention. An email reminder will be sent at 1-month post-intervention. The primary outcome is decisional conflict. Secondary outcomes include measures of readiness and self-efficacy to take action, hearing-related quality of life and empowerment, assessment of the value and impact of *HearChoice*, work performance and health, and feasibility measures. Primary analysis will compare outcomes between *HearChoice* and the active control at the primary endpoint.

**Ethics and dissemination:**

The study was approved by the Curtin University Human Ethics Committee (HRE2023-0024). All participants will provide written informed consent prior to participation. A broad dissemination plan of the study findings includes peer-reviewed publications, scientific conference presentations, articles and presentations for the wider community and public written in lay and accessible language, and social media.

**Trial registration number:**

Australian New Zealand Clinical Trials Registry (ACTRN12624001139561).

**STRENGTHS AND LIMITATIONS OF THIS STUDY**

- The online nature of the randomised controlled trial (RCT) will enable Australia-wide participation and access to a large number of people.
- The online, rather than clinic-based, approach is more likely to recruit those at the early stages of their hearing journey.
- The RCT will evaluate both the effectiveness and health economics of decision aids for hearing difficulties.
- The online nature of the RCT and intervention may not be accessible to all demographic groups and may not be representative of the general population.
- Participants will not be clinically diagnosed with hearing loss (ie, using pure-tone audiometry) as part of the study.

## Introduction

### Background and rationale

 One in seven Australian adults has hearing loss (approximately 3.95 million), which is estimated to increase to 7.78 million by 2066.^1^ The negative impacts of hearing loss are pervasive, impacting communication, interaction with others and social engagement, leading to overall poorer health and well-being,^2^ as well as reduced employment, income, advancement and well-being in the workplace.^3^ Additionally, hearing loss in midlife is a primary potentially modifiable risk factor for dementia.^4^ Despite hearing aids being clinically effective,^5^ only one in three people who would benefit from hearing aids have them.^6 7^ There is a significant time delay of 8.9 years, on average, from becoming aware of hearing difficulties to obtaining hearing aids.^8^ Additionally, the majority of working-aged adults aged between 26 and 64 years, and many who are retired, are not eligible for fully or partially subsidised hearing healthcare through the Australian Hearing Services Programme (HSP). These barriers and low uptake of hearing healthcare contribute to the economic cost of untreated hearing loss to the Australian economy reported at $A 41 billion in 2019–2020.^1^

Low uptake of hearing healthcare is common, with many people not seeking help for hearing problems.^9^ This issue was highlighted by the HSP review published in 2021 that found 60% of those eligible for the HSP do not engage with it.^10^ Understanding the reasons for low uptake of hearing healthcare early in the hearing journey is important to reduce the burden of untreated hearing loss in Australia. The HSP review reported barriers including poor understanding of the impacts of hearing loss, inappropriate expectations about hearing aids, and a lack of information, or misinformation, about hearing loss, hearing aids and other rehabilitation options. For example, a large proportion of people do not believe hearing aids provide benefits.^10^

Part of this lack of understanding may arise from there being little information offered about alternative options available (eg, range and function) for hearing loss, beyond commonly prescribed hearing aids.^11^ Alternative options include technological or non-technological options, for example, over-the-counter self-fitting hearing devices and ‘hearables’,^12^,^14^ assistive listening devices,^15^ communication training,^16^ educational support^17 18^ and auditory-cognitive training.^19^ In addition to the lack of information about the options, client preferences are often not explored, and there is limited information about available hearing healthcare pathways, how to access them and the available options.^20 21^ Lack of information coupled with minimal consideration of personal preferences reduces people’s capacity for informed decision-making, limiting their choice and control over their personal hearing healthcare.^21^ This can lead to reduced help-seeking and action, which is seen in a substantial proportion of those who seek help initially but do not subsequently follow through with decision-making.^22^

Helping a person make informed decisions is a central tenet of audiological practice and person-centred care more generally.^23^ To promote decision-making and help-seeking behaviours, it is necessary to increase capability (eg, recognising hearing difficulties, knowing where to go for help), opportunity (eg, support in decision-making and having access to good-quality information) and motivation (eg, understanding the importance of managing hearing loss).^9^ As such, the research is underpinned by the COM-B model, a framework for understanding behaviour change that proposes behaviour (B) involves three key factors: capability (C), opportunity (O) and motivation (M).^24^ More recently, a comprehensive framework of barriers and enablers to help-seeking and informed decision-making based on the COM-B model has been developed as part of the *HearChoice* research.^21^

Informed decision-making has six key elements: situation diagnosis, choice awareness, option clarification, discussion of harms and benefits, deliberation over patient preferences and making the decision,^25^ all required by the International Patient Decision Aid Standards (IPDAS; http://www.ipdas.ohri.ca/). Decision aids have a positive impact in general healthcare by improving knowledge, informed choice and decision-making.^25 26^ The development of patient decision aids has been highlighted as a research priority in the Australian HSP review^10^ and Roadmap for Hearing Healthcare^27^ and the UK NICE Guidelines for Hearing Loss.^28^ However, evidence-based co-designed decision aids based on IPDAS criteria are limited.^29^

Supporting patients in decision-making through education, and promoting client readiness and motivation in their hearing healthcare, is likely to reduce the delay between awareness of hearing difficulty and seeking help.^10^ *HearChoice*, a newly developed online, modular, interactive and individualised decision support intervention, seeks to tailor this support to close the gap and reduce the identified barriers to hearing healthcare.^21^

### Objectives

This randomised controlled trial (RCT) aims to:

1. Evaluate the effectiveness of the *HearChoice* intervention, as compared against a hearing health option grid, in terms of improving decisional conflict (primary outcome), and a range of secondary outcomes.
2. Perform an economic analysis of *HearChoice* in adults seeking help for their hearing loss.
3. Assess the acceptability and feasibility of *HearChoice* in facilitating decision-making in the target population.

## Methods and analysis

### Trial design and setting

This is a two-arm superiority parallel RCT with an active control. The trial will compare the *HearChoice* decision support intervention to a modified Hearing Loss Option Grid originally developed in the UK based on a qualitative study^20^ and adapted for use in Australia (online supplemental appendix 1).

This article outlines the protocol (V.1.0, 20.2.25) of the *HearChoice* (RCT), which meets the Standard Protocol Items: Recommendations for Intervention Trials (SPIRIT) guidelines.^30^ The SPIRIT Checklist is shown in online supplemental file 2.

The trial was prospectively registered with the Australian New Zealand Clinical Trials Registry. This registration includes all items from the WHO Registration Data Set (V.1.3.1).^31^

**Figure 1:**
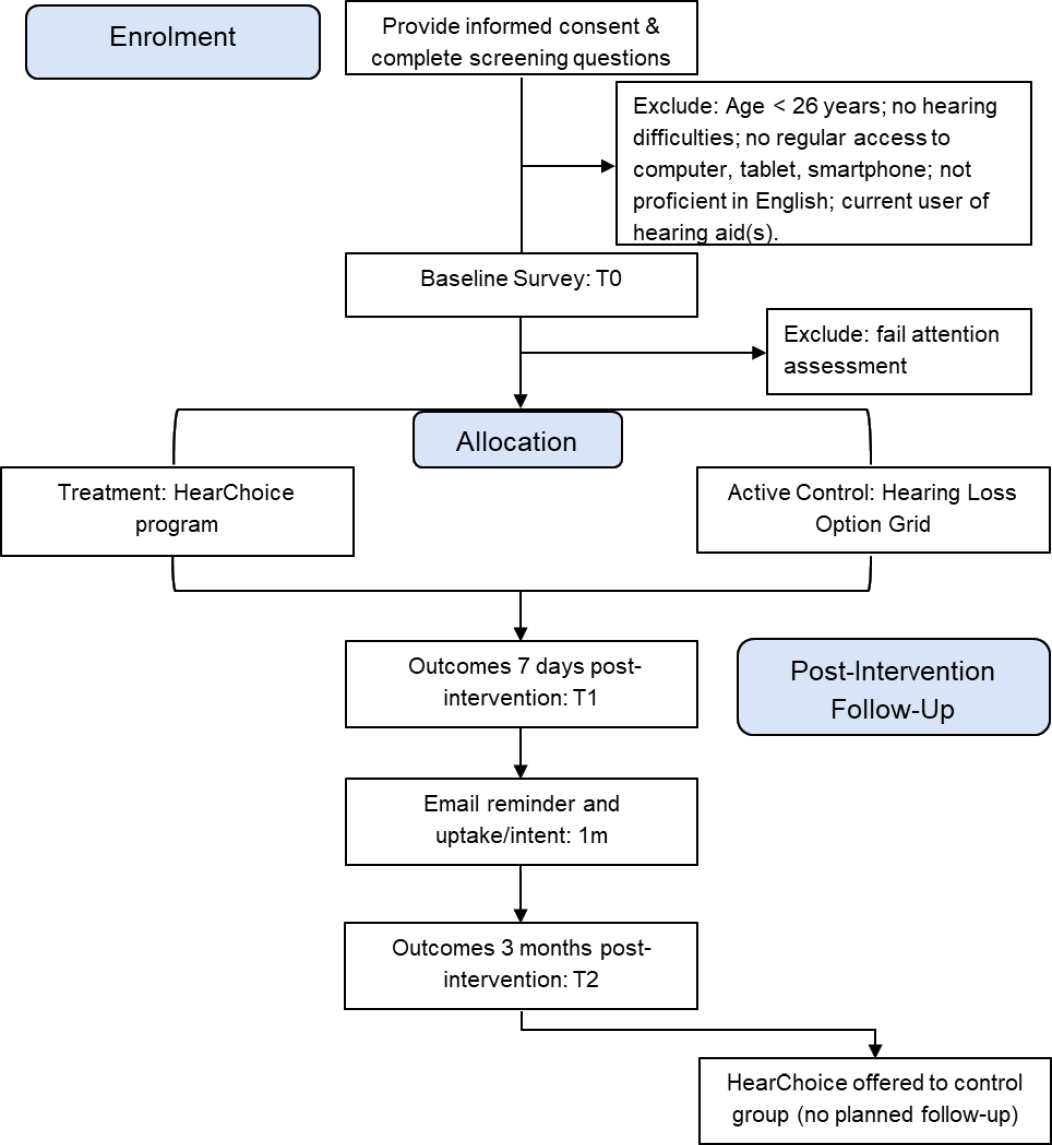
Flow diagram of enrolment, interventions and measures.

The online trial will be conducted by the Behavioural Insights Team (BIT) on Predictiv, their online experiment platform. Participants will be recruited through Pureprofile, a global research panel, who will recruit participants across Australia and send them a link to the trial. All aspects of the trial (eg, informed consent, decision tool interventions and measures) will be delivered online. Participants in both arms will follow the same study schedule, with outcome measures at baseline (T0), 7 days post-intervention (T1, primary endpoint) and 3 months post-intervention (T2) (see figure 1). Participants will be paid participation fees by Pureprofile for completing each of the T0, T1 and T2 surveys. Participants will be paid the same regardless of the intervention arm to which they are allocated. The study sponsor is Curtin University, Perth, Australia, who is responsible for the conduct of the study.

### Participants

#### Eligibility criteria

The inclusion criteria are: (1) aged 26 years and older, (2) subjective experience of hearing difficulty, (3) currently residing in Australia, (4) regular access to a computer, tablet or smartphone to navigate websites and (5) a good understanding of English. The exclusion criterion is current users of hearing aid(s).

#### Sample size

We aim to recruit a sample of 640 participants at randomisation. This sample size is based on a required sample of 352 at T1 and a rate of 45% attrition between T0 and T1. This rate of attrition is typical for studies completed using the Predictiv platform.

The required sample size at T1 was calculated as necessary to detect a moderate effect size of 0.3 at 80% power and an alpha level of 0.05 in a two-tailed test. This moderate effect size was selected pragmatically as there are no published RCTs of decision aids for hearing interventions. Review of previous RCTs of patient decision aids for other, mainly chronic, conditions using the Decisional Conflict Scale revealed a wide range of reported effect sizes (0.03–3.94).^32 33^ The selected effect size of 0.3 is slightly smaller than that reported in a previous study of a similar online decision aid.^34^

### Interventions

#### Treatment group: HearChoice programme

**Figure 2:**
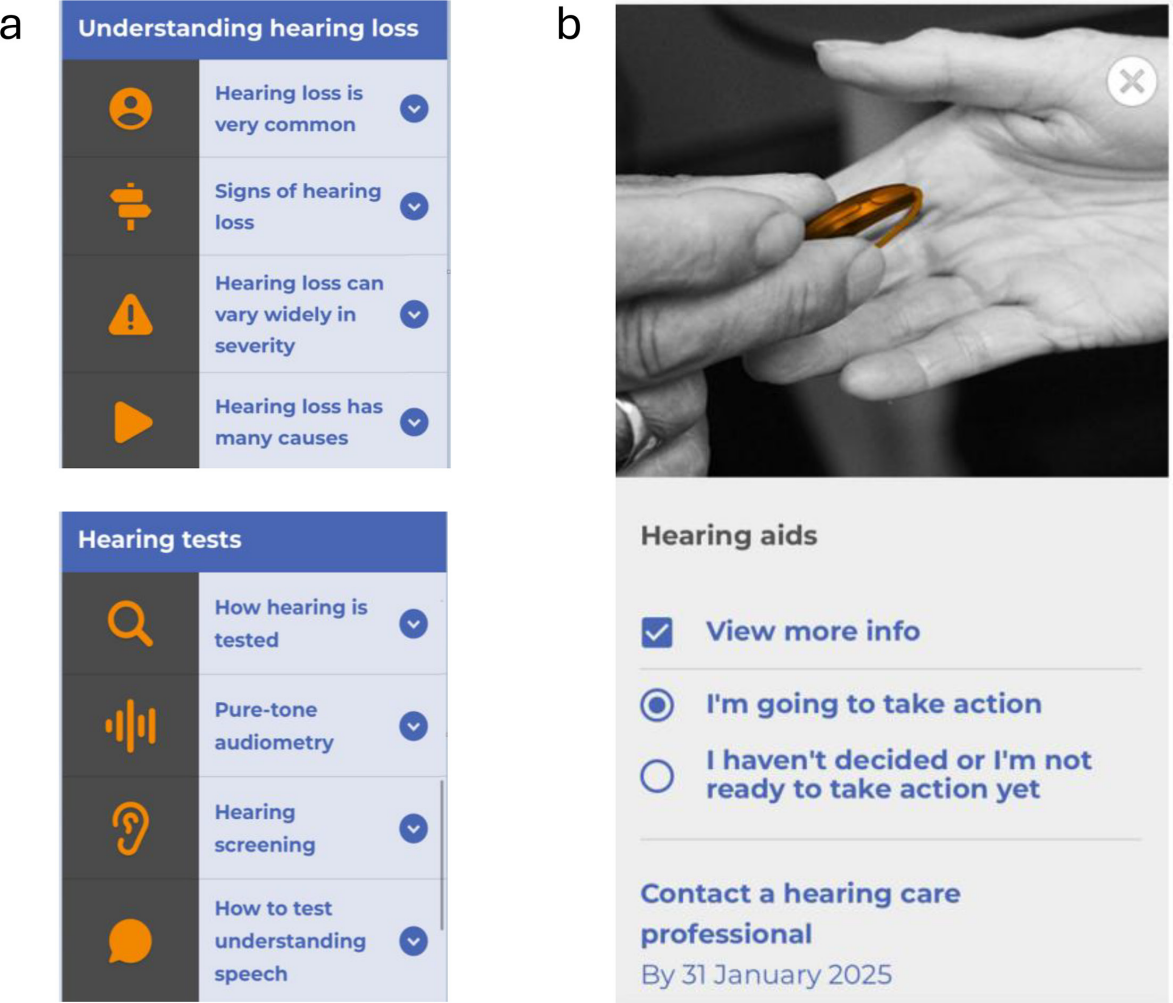
Exemplar screenshots of HearChoice. (**a**) Information delivered via expandable boxes (Learn, Services) and (**b**) taking action for a preferred option (My Plan).

*HearChoice* is a web-based app that has been co-designed through an iterative, participatory approach, based on design thinking principles, with adults living with hearing loss, advocacy groups and hearing care professionals (HCPs).^21^ *HearChoice* is an interactive patient decision-support intervention for adults with hearing difficulties comprising six sections (About, Learn, Services, Choice, My Plan, Resources). Participants will independently access *HearChoice* via their smartphone, tablet or computer by following a link via a unique identifier number. *HearChoice* is designed to provide a step-by-step guide to decision-making about hearing healthcare, including background information about hearing loss, the importance of management, and guidance about hearing healthcare options. *HearChoice* is presented using written text, images, self-report questionnaires, an online hearing test, videos of personal experiences of adults with hearing difficulties, and a set of relevant resources (eg, technology levels and costs for hearing aids and direct-to-consumer products, implantable devices, tinnitus). Information is presented via expandable boxes grouped under sub-headings (figure 2). Participants can choose where to go and what to engage with, although it is recommended that they follow the flow through the sections from About to My Plan, where they make their decision. The six sections are:

1. *About*. Explains what *HearChoice* is, its aim and how to use it.
2. *Learn*. Three subsections on evidence-based information on hearing loss and management: understanding hearing loss; impact of hearing loss and benefits of hearing healthcare; ‘Mythbusters’ to dispel the myths about hearing loss and management.
3. *Services*. Four subsections about the Australian hearing healthcare provision: questionnaire to assess eligibility for Australian government support; healthcare professionals and services; hearing tests, including screening and speech understanding; an online digit triplet test in noise hearing test, and the 5-item Social Isolation Measure to assess psychosocial aspects of hearing loss.^35^
4. *Choose*. Three subsections offer a broad range of 11 hearing healthcare options: values clarification questionnaire (what matters most for you to hear well?); non-device options comprise five person-centred options including ‘Do Nothing’; technology options comprise six options including ear-level and remote hearing devices. For each option, provision and costs are described. Participants can choose the preferred options that will meet their needs, which are added to their My Plan.
5. *My Plan*. Using an interactive format, participants can compare their chosen options and obtain more in-depth information about options, including pros and cons. Participants are prompted to decide whether to take action or not. Potential actions, such as seeing an HCP or accessing an online programme, are presented which they can select, along with selecting a date by which to complete the action. The ‘My Plan’ page is built, providing a tailored description of hearing choice options that suit their preferences and values. This can be saved or printed for future reference (eg, to take to an appointment with an HCP).
6. *Resources*. A list of 14 sets of resources about hearing healthcare (eg, medical advice; understanding hearing aid technologies, including costs and technology levels; hearing market), special populations and conditions (eg, hearing loss and dementia; tinnitus; cochlear implants) and information About *HearChoice*.

*HearChoice* adheres to the IPDAS guidelines,^36^ meeting all six qualifying criteria confirming that *HearChoice* is a valid decision aid tool. All 10 IPDAS Certification criteria are met, indicating a high-quality and evidence-based decision aid and suggesting trustworthiness, usability and effectiveness in informed-decision-making relating to hearing health. The development of *HearChoice* was underpinned by behaviour change theory^24^ and implementation science,^37^ addressing barriers and enablers to help-seeking and decision-making.^21^

#### Active control Group: Option Grid

A Hearing Loss Option Grid (a one-page PDF) that met IPDAS guidelines^36^ was developed for the Australian hearing healthcare system, based on an existing Option Grid developed in the UK (online supplemental appendix 1). The Option Grid was designed to provide a framework for decision-making about technology-based hearing management options, presented using written text. The option grid included three options (hearing aids, assistive listening devices and do nothing), and information for each option about what this involves, expectations, benefits, cost and extra considerations such as maintenance.

#### Criteria for discontinuing or modifying allocated interventions

There are no a priori criteria for discontinuing or modifying allocated interventions. Participants will be informed that they are able to withdraw participation at any point in the study without negative consequences for their hearing healthcare. They do not have to give a reason for withdrawal.

#### Strategies to improve adherence to interventions

Participants can access their allocated intervention throughout the 3-month period of the trial by either bookmarking (*HearChoice*) or downloading (Option Grid) the intervention. Clear information will be presented to explain how this can be achieved. The 7-day period between allocation of the intervention (T0) and the primary endpoint (T1) will allow participants time to work through the content at their own pace and spend as little or as long as they choose. At T1 and T2 timeframes, the intervention will be offered again within the survey, with the option to bookmark or save. Similarly, the intervention will be re-presented at the 1-month timepoint in an email sent to nudge the participant that also asks them to think about their options, what actions they have taken and what actions they intend to take.

#### Relevant concomitant care permitted or prohibited during the trial

There will be no restrictions regarding concomitant care during the trial outside of the trial arms. Similarly, there will be no restriction on using other hearing-related websites, although participants will be asked to report whether they did so. Information regarding any action taken towards seeking hearing healthcare will be obtained at all follow-up data collection points (T1, 1m, T2).

#### Provisions for post-trial care

Participants in the *HearChoice* group will be receiving the best available evidence of a broad range of options available for dealing with hearing difficulties. Those allocated to the active control group will be offered access to the *HearChoice* website at the conclusion of the 3-month follow-up period.

### Outcomes

Delivery of primary and secondary outcomes via an online survey is shown in table 1. Participants will be allowed to complete the survey up to 7 days following its delivery at T0, and 10 days for T1, 1m and T2. All questions, if applicable, are mandatory to enable progression through the survey. Reminders will be sent to facilitate survey completion: for T0, one reminder 2 days following initial survey delivery, and for T1 and T2, reminders at 2 days and 7 days following initial surveys.

**Table 1:** Summary of outcomes, instruments and timepoint for administration at baseline (T0), 7 days post-intervention (T1) and 3 months post-intervention (T2)

Outcome	Instrument	Baseline (T0)	7 days (T1)	3 months (T2)
	**Effectiveness outcomes**			
Primary outcome				
Decisional conflict	Decisional conflict Scale	X	X	X
Secondary outcomes				
Readiness for hearing healthcare	Ida Institute Line 1	X	X	X
Self-efficacy for hearing healthcare	Ida Institute Line 2	X	X	X
Hearing-related quality of life	Revised Hearing Handicap Inventory—Screening	X		X
Empowerment	Empowerment Audiology Questionnaire—15 items	X	X	X
Intention to take action	Bespoke project-related question		X	X
Preparedness for decision-making	Preparation for Decision-making Scale		X	X
Impact of the decision tool	User-Mobile Application Scale—User		X	X
Action taken	Bespoke project-related question		X	X
Benefit of hearing aids	Glasgow Hearing Aid Benefit Profile Part 2			X
Economic benefit	WHO Health and Work Performance Questionnaire	X		X
	**Feasibility and implementation**			
Acceptability	Acceptability, usability, usefulness, satisfaction, accessibility		X	X
Consent rate	Completion at T0 vs number of those eligible	X		
Attrition	Completion at T1 vs T0	X	X	
Adoption	Uptake of intervention (treatment and control)	X	X	X
Fidelity (adherence)	Website analytics (throughout study)	X	X	X

To ensure high quality data, participants will be asked to complete an attention check at all time points prior to the outcome measures. The attention check is to identify whether participants can read and respond correctly to a simple instruction to minimise compromising data quality. Participants will be asked to indicate that they have read ‘Moderately interested’ and ‘Slightly interested’ in a sentence. Participants who fail the check at T0 will be excluded from the trial. Participants who fail at the other time points will be able to continue with the survey, but their failure of the attention check will be noted and potential impacts will be evaluated in the analysis.

#### Primary outcome

The primary outcome, decisional conflict, is measured by the 16-item Decisional Conflict Scale.^38^ Decisional conflict is the uncertainty a person experiences when faced with a choice that involves risk, loss, regret or challenges to personal values. In this study, the choice relates to hearing healthcare. There are five subscales (Informed, Values Clarity, Support, Uncertainty, Effective Decision).

The *HearChoice* intervention will be considered effective if the estimated effect on this outcome is considered statistically significant (p<0.05) and clinically significant (Cohen’s *d*=moderate (0.3) or greater) for the overall score.

#### Secondary outcomes

Patient-reported outcome measures to assess secondary outcomes relevant to the clinical effectiveness evaluation are described. A feasibility study was not conducted prior to the RCT; therefore, feasibility and implementation data will be collected.

- *Ida Institute Readiness to Take Action (Line 1)*,^39^ visual analogue scale, 0–10.
- *Ida Institute Self-efficacy to Take Action (Line 2)*,^39^ visual analogue scale, 0–10.
- *Revised Hearing Handicap Inventory-Screening*,^40^ validated, 10 items, 3-point Likert Scale (yes, sometimes, no).
- *Empowerment Audiology Questionnaire*,^41^ validated, 5 items, 5-point Likert Scale (strongly disagree to strongly agree, not applicable).
- *WHO Health and Work Performance Questionnaire*,^42^ validated, adaptive subset of questions on paid/unpaid work.
- *Intention to take action*, 11 healthcare options, contact HCP, family doctor or hearing support organisations. This question will also be delivered at the 1m email reminder.
- *Preparedness for Decision-making*, validated,^43^ 10 items, 5-point Likert scale (not at all to a great deal).
- *User-Mobile Application Rating Scale*,^44^ validated, subset of 6 items for perceived impact, 5-point Likert scale (strongly disagree to strongly agree).
- *Action taken*, 11 healthcare options, contact HCP, family doctor or hearing support organisations. This question will also be delivered at the 1m email reminder.
- *Glasgow Hearing Aid Benefit Profile*,^45^ validated, four items (use, benefit, residual disability, satisfaction) across four pre-defined situations, 5-point response specific to the situation and item (0=100%). Only for those who take up hearing aids.
- *Feasibility* (acceptability, usability, usefulness, satisfaction, accessiblity), 10 items, 5-point Likert scale (not very much to very much so).
- *Consent rate*, completion of outcomes at T0 compared with those who are eligible (%).
- *Attrition rate*, completion of outcomes at T1 compared with T0 (%).
- *Adoption*, uptake of intervention (*HearChoice*, Option Grid) across the study.
- *Fidelity*, time spent adhering to the allocated intervention across the study (s).

#### Other sociodemographic and clinical measures

Measures of demographic, hearing and characteristics of the intervention and control groups will be collected at T0, which include: gender, Australian residency, eligibility for governmental assistance in hearing, Aboriginal or Torres Strait Islander status, Australian state or territory residence, annual household income, employment status, educational status, marital status, duration of hearing difficulty and subjective level of hearing ability, hearing test(s) previously conducted, digital literacy, English literacy, visual correction/difficulty and other health conditions. Dimensions of the device (eg, smartphone, tablet, laptop) that the participants use will be collected.

### Assignment of interventions

#### Sequence generation

Simple randomisation will be used, with the aim of obtaining a ratio close to 1:1. With large sample sizes, simple randomisation should allow us to reach the same allocation as other, more complex methods, such as block randomisation. Participants will be assigned a random number (1 or 2), corresponding to the relevant trial arm. This will be done on the Predictiv platform when participants enter at T0. The randomisation uses a sequence of pseudo-random numbers generated using a Mersenne Twister Random Number Generator, and the numbers are automatically stored in the data on Predictiv. There will be no restrictions (eg, blocking) on the randomisation.

#### Allocation concealment and blinding

Randomisation will occur via a remote online central randomisation system via the Predictiv platform. Participants cannot access their survey data and are blinded to their group allocation. At the end of T1, we will ask participants to say which intervention arm they thought they had been allocated. The researchers will also be blinded to randomisation and group allocation, where possible.

### Data collection, management and analysis

#### Data collection and management

All data collected by BIT will be stored on BIT’s Google Drive compliant with ISO 27001,^46^ as well as the secure, password-protected Predictiv platform that only project team members can access. Personal email data collected at T1 to enable contact for the 1-month timepoint will be deleted at the end of the trial period. Data will be cleaned for test responses, duplicate hashed IP addresses (that limit the risk of individual identification), participant IDs and other unique identifiers related to the participant’s device. The cleaning code will be quality-assured by an independent researcher at BIT. Data will be sent securely to researchers at Curtin University and stored within a secure data storage drive based at Curtin University, Perth, Australia. This secure location is designed for storage of sensitive information and is the default storage option for research data generated at Curtin University. Data will be retained for a minimum of 7 years. A data management plan has been developed and approved by all partners involved in the RCT to detail data management procedures.

An attrition rate of approximately 45% between T0 and T1 is anticipated based on similar online studies.^47 48^ To promote participant retention and completion at T1, the duration from T0 to T1 is set at 7 days to give participants time to use the interventions in a timely manner and consider their hearing health needs and decision-making for the available options. We considered this time period sufficiently long to observe the intervention’s effects yet short enough to avoid significant participant attrition. The 1-month reminder email will be delivered as a behavioural ‘nudge’ in terms of intention and action to uptake hearing health. The allocated interventions will be made available to the participants throughout the trial. Interventions can be downloaded or bookmarked, and device-specific instructions will be provided to facilitate this. Additionally, the allocated intervention will be re-presented at T1, 1m and T2.

#### Confidentiality

Reidentifiable personal information will be collected and stored on password-protected secure servers at BIT and Curtin University with a two-step authentication process, restricted access to BIT and key members of the research team (MF, EB). All participants will be assigned a unique code and their de-identified data will be stored with this code. A separate document linking participant codes to their identity will be accessible only to Authors MF and EB. If requested, data will be made available to the sponsor, human ethics committee and national regulatory bodies. Confidentiality will be ensured before, during and after the trial through these measures.

#### Data monitoring

Data collection will be regularly monitored by researchers at BIT. This will include a pilot soft launch to ensure data are correctly collected and the survey is working from a technical perspective. Any required changes will be made. BIT will work with Pureprofile to monitor traffic to the survey, attrition and other issues that might arise. Any issues will be raised with the chief investigator (MF) and decisions made in consultation with the research team, as required.

Any adverse event will be immediately reported to the chief investigator, who will report the deviation to the Curtin University Human Research Ethics Office.

#### Statistical methods

A full statistical analysis plan will be finalised prior to data lock. No interim analyses to stop for efficacy or futility are planned.

Demographic and hearing characteristics of the intervention and control groups will be described at T0. For the primary and secondary outcome measures at each timepoint (T0, T1, T2), we will provide the mean and 95% CIs by group. Differences between groups will be explored at each timepoint using independent samples t-tests, with correction for Type I errors as required. The primary analysis will evaluate whether there is a difference between the intervention and control groups at the primary study endpoint T1.

Analysis of variance will be used to compare change over time on each outcome measure between the intervention and control groups. Additional analyses will be performed to explore the impact of demographic, hearing-related characteristics and intervention engagement on the outcome measures over time between groups.

Economic analysis involves estimation of societal costs and costs associated with the development and use of the decision aid, and costs of fitting hearing aids or equivalent. Additionally, the impact of the intervention of work will be explored on both employment status (focusing on hours of work per week), and measures of absenteeism and presenteeism, specifically the WHO Health and Performance Questionnaire.^42^

Acceptability and feasibility data will be summarised by descriptive statistics and compared within and between intervention groups. This will include a flow diagram of the numbers of participants across the trial as indicated in figure 1 (eg, number screened, eligible, completion at each timepoint). For the *HearChoice* group, additional analyses using website analytics on participants’ engagement with *HearChoice* across the trial period will be performed based on content viewed. These results, along with those from the evaluation outcome measures, will help inform future trials of decision aids in hearing healthcare.

After completion of all planned analyses, data will be made available on reasonable request and on signing a unilateral data sharing agreement.

### Patient and public involvement

Patient and public involvement is an integral part of the research, following good practice principles.^49^ The *HearChoice* Consumer Advisory Group was involved in commenting and editing participant-focused written materials, and along with the Consumer and Community Involvement panel, contributed to the co-development of the *HearChoice* app.

## Ethics and dissemination

### Ethics

The research study has been approved by the Curtin University Human Ethics Committee HRE2023-0024. Following presentation of the online Participant Information Sheet, informed consent will be indicated online by agreeing to a series of five statements to ensure the participant has understood the project (online supplemental appendix 2). Any protocol amendments to the protocol will be submitted to the Curtin HREC for approval, and the ANZCTR clinical trial registration will be updated. Anonymised data will be made available on completion of publication of the full programme of research on signing a unilateral data sharing agreement and will be made available for 5 years.

Financial and other competing interests for the principal investigators for the trial will be documented as required.

### Dissemination

The trial and analysis will be reported according to the Consolidated Standards of Reporting Trials.^50^ Trial results will be communicated to healthcare professionals, researchers, the public, advocacy groups and other relevant groups through a combination of: international peer-reviewed publications, professional and patient advocacy publications and newsletters, blogs and social media, institutional websites of the research team, National and Health Medical Research Council and Australian Department of Health websites and bulletins, oral and poster presentations at scientific conferences and other public and wider community forums (online, in person), and a newsletter to participants interested in hearing about the results of the research. Note: all public-facing materials, whether written or verbal, will be presented in lay and accessible language with input from the project’s Community Advisory Group.

Authorship in peer-reviewed publications will be guided by the International Committee of Medical Journal Editors. Contributors who do not meet these criteria will be acknowledged in the acknowledgement sections of the paper. We do not intend to use professional writers.

## Discussion

There are major barriers to help-seeking and decision-making in hearing healthcare.^21^ These barriers lead to many adults with hearing loss not knowing how to seek help for their hearing, and not being aware of the broad range of available hearing healthcare options, leading to uncertainties as to how to make decisions about which options to choose. These barriers early in the hearing health journey result in many people living with untreated hearing loss and its consequences, such as increased risk of dementia, negative impact on ability to perform work duties, decreased opportunity for social interaction and subsequent effects on well-being.^20^,^22^ There is a paucity of validated decision tools and research to support their effectiveness, as indicated by the research priorities in Australia and the UK.^10 27 28^ The *HearChoice* research project, including this RCT, aims to address these issues by identifying the benefits of the *HearChoice* app, and its impact on the individual, including their employment. This will establish the value of *HearChoice* in terms of hearing healthcare and economic benefits. The *HearChoice* intervention will be the first comprehensive online decision support intervention co-developed for public use in Australia and evaluated with an RCT, with plans for further global reach. The *HearChoice* app will be made available online for support early in the hearing health journey, and for hearing healthcare professionals to use with clients to facilitate shared decision-making in clinics. We anticipate these benefits will result in a greater number of empowered individuals having better informed choice and control over their hearing healthcare.

There are a number of strengths and limitations to this study. This is the first RCT to evaluate the effectiveness of an online decision aid used in audiology, addressing research priorities set by key stakeholders (eg, healthcare professionals, patients).^10 27 28^ The online nature of the RCT takes a population approach that will enable Australia-wide participation and recruit participants across a wide range of hearing health journey stages. This will include those who are in the early stages of their hearing journey, who often do not present clinically (the average delay from noticing hearing difficulties to obtaining hearing aids is 8.9 years).^8^ Thus, this online trial design will help inform how the HearChoice app might be delivered and implemented in future.

The online nature of the RCT also has limitations. It may not be accessible to all demographics with hearing difficulties, in particular those who are not digitally literate and do not have access to the internet (ie, the ‘digital divide’),^51^ therefore the trial sample may not be representative of the general population. The lack of a clinical diagnosis of hearing loss (ie, by pure-tone audiometry) is a limitation, and we are reliant on self-report of hearing difficulties. However, two systematic reviews have shown that self-reported hearing difficulties are a significant factor in help-seeking behaviours and hearing aid uptake.^52 53^ The self-report of hearing difficulties may result in people over-estimating or under-estimating their hearing abilities, resulting in the RCT including some people who may not be suitable for hearing healthcare, and the inverse, whereby some people with hearing care needs who are initially approached are not identified as eligible for the trial. To address the former, further examination of the hearing difficulties will be assessed using the validated Revised Hearing Handicap Inventory-Screen^40^ and a simple baseline self-assessment of overall hearing based on a 10-point visual analogue scale (1=poor, 10=excellent).

In summary, this RCT will provide effectiveness, economic and feasibility results to guide implementation of *HearChoice* and provide valuable insights into the impact of the *HearChoice* decision aid that is designed to educate, engage and empower those with hearing difficulties.

## Supplementary material

10.1136/bmjopen-2025-106751 online supplemental file 1

10.1136/bmjopen-2025-106751 online supplemental file 2
