# Supplementary material for: Evaluation of a digital health decision intervention to support management decision-making for adults with hearing loss: protocol for the HearChoice randomised controlled trial
Source: BMJ Open. 2025 Oct 23;15(10):e106751. doi: 10.1136/bmjopen-2025-106751 (PMC12551472; doi:10.1136/bmjopen-2025-106751)
Supplement: online supplemental file 1 [file bmjopen-15-10-s001.docx]

**Supplementary Digital Content**

**Evaluation of a digital health decision intervention to support management decision-making for adults with hearing loss: Protocol for the HearChoice randomised controlled trial**

Appendix 1: Option Grid for Hearing Loss: Hearing Technology Options

Appendix 2: Consent statements

**
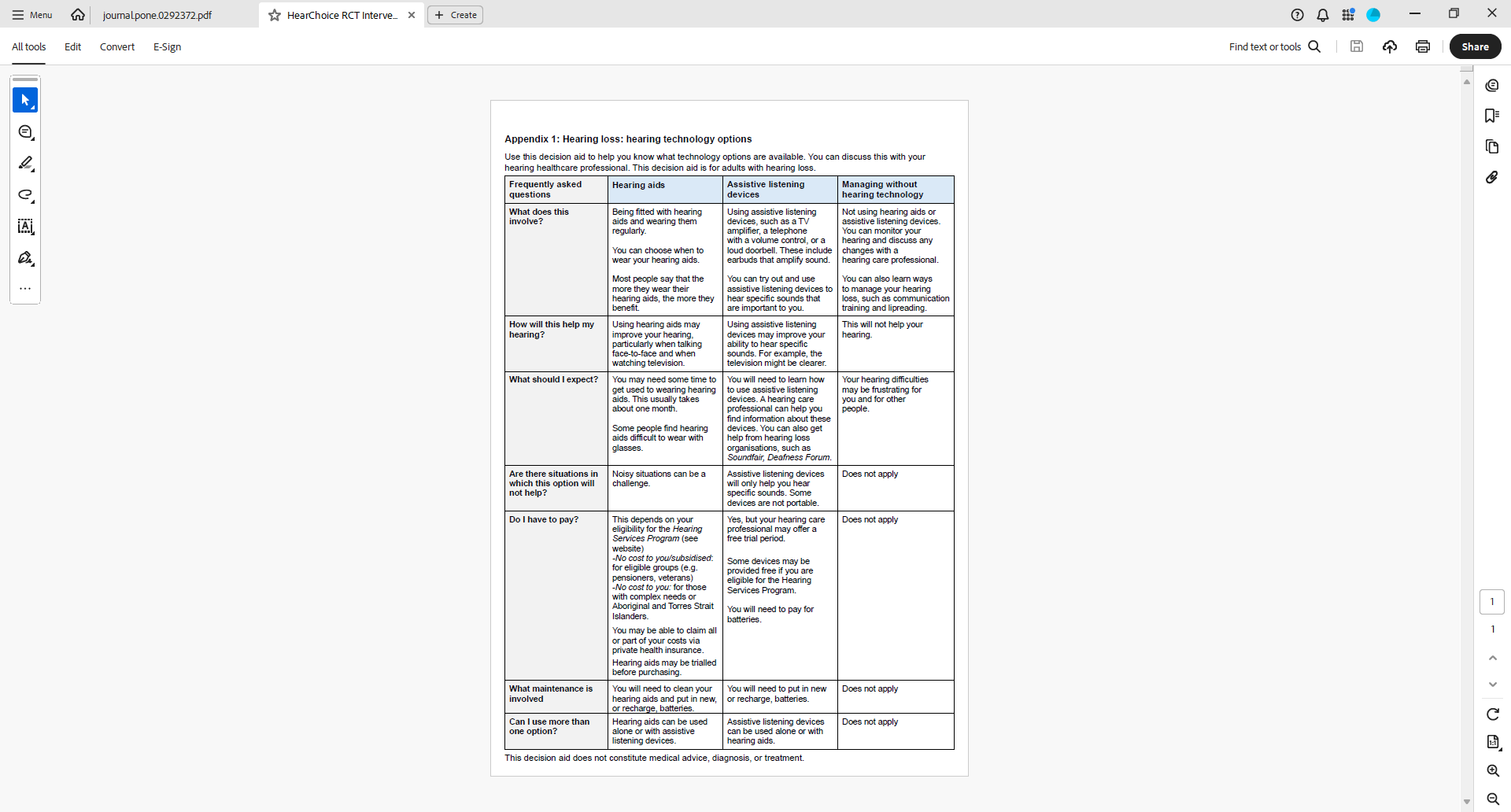
**

**Appendix 2: Consent statements following the online Patient Information Sheet**

- I have read the information statement version listed above and I understand its contents.
- I believe I understand the purpose, extent and possible risks of my involvement in this project.
- I voluntarily consent to take part in this research project.
- I have had an opportunity to ask questions and I am satisfied with the answers I have received.
- I understand that this project has been approved by Curtin University Human Research Ethics Committee and will be carried out in line with the National Statement on Ethical Conduct in Human Research (2007).
