# Supplementary material for: Evaluation of a digital health decision intervention to support management decision-making for adults with hearing loss: protocol for the HearChoice randomised controlled trial
Source: BMJ Open. 2025 Oct 23;15(10):e106751. doi: 10.1136/bmjopen-2025-106751 (PMC12551472; doi:10.1136/bmjopen-2025-106751)
Supplement: Supplementary file 3 [file bmjopen-2025-106751.reviewer_comments.pdf]

PEER REVIEW HISTORY

BMJ Open publishes all reviews undertaken for accepted manuscripts. Reviewers are asked to complete a checklist review form and are provided with free text boxes to elaborate on their assessment. These free text comments are reproduced below.

ARTICLE DETAILS

Title (Provisional)

Evaluation of a digital health decision intervention to support management decision-making for adults with hearing loss: protocol for the HearChoice randomised controlled trial

Authors

Ferguson, Melanie; Sherman, Kerry A.; Bothe, Ellen; Timmer, Barbra; Dawes, Piers; Myers, Bronwyn; Norman, Richard; Mejia, Jorge; Bennett, Rebecca J.; Mottershaw, Abigail L.; zu Brickwedde, Elena Meyer; Convery, Elizabeth; Gyani, Alex

---

VERSION 1 - REVIEW

**Reviewer**	**1**
**Name**	**Robler, Samantha**
**Affiliation**	**University of Arkansas for Medical Sciences**
**Date**	**16-Aug-2025**
**COI**	**None**

Evaluation of a digital health decision intervention to support management decision-making for adults with hearing loss: Protocol for the HearChoice randomised controlled trial

This study protocol is for an online RCT that started recruitment on 29th May, 2025 to evaluate the effectiveness, economic benefit, and feasibility of an online decision tool called HearChoice for those entering the hearing care journey. The goal is to recruit 640 participants through November of 2025. This is a well-written protocol and I commend the authors for their contribution to improving the patient care journey for those touched by hearing loss. I have minor suggestions to enhance the readability of the submitted protocol.

Line-by-line suggestions:

Page 7, line 51. Consider better defining COM-B model, e.g. behaviour system' involving three essential factors: capability, opportunity, and motivation (COM-B). Also include a reference.

Page 8, starting line 36. I recommend presenting the primary and secondary aims more clearly. I understand there are a number of secondary aims; however, it is a bit confusing to list some but not all secondary outcomes, particularly when you look across table 1, the trials registry, the outcomes section and how analyses are presented. Consider trying to present them similarly. One option may be to pull out or collapse economic outcome between the text/table.

Page 9, line 41. “informed 294” is not clear; please clarify, define, or correct, if typo.

Page 10, lines 49-56. Consider including any publications surrounding the co-design and development of HearChoice.

Page 14, line 49. Define “attention check”

Page 15, line 11. Define “decisional conflict”

Page 15, starting line 27. I defer to the journal, but the format of the scales listed as they are is a bit hard to read. Perhaps a bulleted indented list or include the scale details within table 1, since they are also listed there.

Page 18, line 51. “At the end of T1, we ask which arm they think they allocated.” Re-write for grammar. Also check tense.

Page 19, line 9. Google Drive for business? Make sure to speak to data security.

Page 19, line 16. Consider defining duplicate hashed IP addresses and use in securing original IP address.

Page 19, line 35. Change “was” to “is”

Page 20-21, lines 52-10. I’m not a biostatistician, but I imagine the analysis would need careful consideration to avoid inflating the chance for type 1 error with performing several independent t-tests. Please note any statistical considerations, if applicable.

Page 22, lines 6-11. Provide a reference, if possible

VERSION 1 - AUTHOR RESPONSE

Reviewer 1

*This study protocol is for an online RCT that started recruitment on 29th May, 2025 to evaluate the effectiveness, economic benefit, and feasibility of an online decision tool called HearChoice for those entering the hearing care journey. The goal is to recruit 640 participants through November of 2025. This is a well-written protocol and I commend the authors for their contribution to improving the patient care journey for those touched by hearing loss. I have minor suggestions to enhance the readability of the submitted protocol.

RESPONSE: Thank you for your very positive comments.

Line-by-line suggestions:
*Page 7, line 51. Consider better defining COM-B model, e.g. behaviour system' involving three essential factors: capability, opportunity, and motivation (COM-B). Also include a reference.

RESPONSE: Page 7 Line 51 in the initial manuscript was referring to the framework we have developed so a reference to that has been added (21) (Page 8 Line 2). To explain the COM-B model, a new sentence has been added (Page 7 Lines 19-22).

*Page 8, starting line 36. I recommend presenting the primary and secondary aims more clearly. I understand there are a number of secondary aims; however, it is a bit confusing to list some but not all secondary outcomes, particularly when you look across table 1, the trials registry, the outcomes section and how analyses are presented. Consider trying to present them similarly. One option may be to pull out or collapse economic outcome between the text/table.

RESPONSE: The current objectives separate the three main type of outcomes: patient-reported outcomes (primary and secondary), economic analysis, and feasibility outcomes. These are now noted in this order in Table 1 for consistency, with the WHO HPQ moved to the end of the secondary outcomes. The costs associated with the economic analysis are not collected from participants, whereas the WHO HPQ is, so is included in Table 1 and the registry. The outcomes are all stated in the registry as per Table 1 with the exception of consent rate and attrition, which are reported as standard in RCTs, and here are specified as part of the feasibility aspect of the trial. As such, we think the objectives, outcomes table and registry are in broad alignment.

*Page 9, line 41. "informed 294" is not clear; please clarify, define, or correct, if typo.

RESPONSE: '294' should have read 'consent' and has now been corrected (Page 9 Line 19). Thank you for spotting this.

*Page 10, lines 49-56. Consider including any publications surrounding the co-design and development of HearChoice.

RESPONSE: The development paper is currently in preparation. We have referred to another publication that refers to the co-design and development of HearChoice (ref 21) (Page 11 Line 5).

*Page 14, line 49. Define "attention check"

RESPONSE: The attention check, and its purpose, has been described (Page 15 Lines 8-11).

*Page 15, line 11. Define "decisional conflict".

RESPONSE: Two sentences provide a brief explanation is given (Page 15 Lines 17-19).

*Page 15, starting line 27. I defer to the journal, but the format of the scales listed as they are a bit hard to read. Perhaps a bulleted indented list or include the scale details within table 1, since they are also listed there.

RESPONSE: The secondary outcomes are now bulleted, which along with the italicised title of the outcomes should make it easier to read. An earlier attempt to add all this information into Table 1 made it too busy and difficult to read (Pages 16-17).

*Page 18, line 51. “At the end of T1, we ask which arm they think they are allocated.” Re-write for grammar. Also check tense.

RESPONSE: The sentence has been rewritten in the future tense. (Page 19 Line 22).

*Page 19, line 9. Google Drive for business? Make sure to speak to data security.

RESPONSE: We have added that the Google Drive is compliant with ISO 27001with citation.

*Page 19, line 16. Consider defining duplicate hashed IP addresses and use in securing original IP address.

RESPONSE: Hashed IP addresses has been clarified with added text “that limit the risk of individual identification” (Page 20 Line 9).

*Page 19, line 35. Change “was” to “is”

RESPONSE: Corrected (Page 20 Line 17). A search for other instances was also checked.

*Page 20-21, lines 52-10. I’m not a biostatistician, but I imagine the analysis would need careful consideration to avoid inflating the chance for type 1 error with performing several independent t- tests. Please note any statistical considerations, if applicable.

RESPONSE: There is only one comparison for the primary outcome. For the secondary outcomes, there are a number of multiple comparisons using the initial planned t-tests and their exploratory nature is now noted, along with the correcting for Type I errors, as required (Page 22 Lines 6-7).

*Page 22, lines 6-11. Provide a reference, if possible

RESPONSE: A sentence stating PPI is good practice along with adding a reference to the MRC Guidance on developing and evaluating complex intervention (Skivington et al 2021) has been added. (Page 23 Lines 6-7).
